# Airway Anesthesia for Awake Tracheal Intubation: A Review of the Literature

**DOI:** 10.7759/cureus.16315

**Published:** 2021-07-11

**Authors:** Piotr Kostyk, Karen Francois, Irim Salik

**Affiliations:** 1 Anesthesiology, Westchester Medical Center, Valhalla, USA

**Keywords:** airway topicalization, airway blocks, awake intubation

## Abstract

Airway topicalization is frequently utilized by anesthesiologists to facilitate open airway procedures, aid intubation for a difficult airway, and prevent adverse respiratory events. This review article summarizes the techniques available for airway topicalization for a patient who is deemed to be difficult to intubate. We focus on the indications for use, local anesthetic maximum dosages and safety profiles, sedation techniques, and trials and pitfalls during airway topicalization for difficult intubation.

## Introduction and background

In the general population, the approximate incidence of Cormack and Lehane laryngoscopy grades 3 and 4 is 10%, difficult intubation is 1%, and difficult bag-mask ventilation is 0.08%-5% [1-2]. A difficult airway can lead to various adverse outcomes, including anoxic brain injury, cardiopulmonary arrest, unanticipated surgical airway, airway trauma, damage to the teeth, hypertension, tachycardia, arrhythmias, or death. Difficult ventilation can be caused by inadequate mask or supraglottic airway seal, excessive resistance or gas leak leading to inadequate chest movement, absent or reduced breath sounds, cyanosis, decreasing oxygen saturation, gastric dilatation, and absent or inadequate exhaled carbon dioxide [1-2]. Basic preparation for difficult airway management includes appropriate ancillary equipment, experienced personnel for assistance, patient education, and thorough preoperative evaluation [3]. An antisialagogue may be recommended to dry secretions for visual field optimization during flexible bronchoscope use. Once airway topicalization has been achieved with or without sedation, a flexible bronchoscope can be passed through the vocal cords into the trachea until the tracheal cartilaginous rings and the carina are identified. Next, an endotracheal tube (ETT) is passed, the cuff inflated, an anesthesia circuit connected, and tube placement confirmed clinically and by capnography. At this point in time, anesthesia can be induced via an intravenous or inhalational technique.

We carried out a PubMed search utilizing the keywords local anesthesia, airway, topical, topicalization, airway anesthesia, laryngeal, endotracheal, difficult airway, aspiration, and lidocaine. Manuscripts retrieved using these search items were reviewed. In addition, references of these papers were examined and papers of relevance were also reviewed.

## Review

Awake tracheal intubation

In theory, the safest technique to secure a difficult airway may be awake tracheal intubation (ATI) although it can lead to significant patient anxiety and discomfort. Appropriate topicalization of the larynx and trachea prior to intubation has been shown to prevent increases in heart rate and arterial blood pressure while also reducing the risk of coughing during anesthetic emergence [4-5]. Although there is not a reliable method for the prediction of difficult airway management, there are some identifying factors for patients who require ATI. Some examples of patients that may pose a problem include those with a history of head and neck radiation, surgery, or malignancies, glottic tumors, unstable cervical spines, limited mouth opening or neck extension, known difficult airways due to previous airway mishaps, airway compromise, and morbid obesity. ATI should be considered in patients with a difficult airway who are appropriately nil per os (NPO), as the potential for aspiration of gastric contents during ATI is still present. Contraindications to ATI include airway trauma, an uncooperative patient, patient refusal, and local anesthetic allergy.

Doyle identifies six key airway management decisions prior to ATI [6]. Primarily, is the airway so challenging that a tracheostomy should be carried out under local anesthesia? If this is not the case, and a supraglottic airway is not ideal, an ATI may be desirable. The practitioner should then decide whether a nasal or oral route is required according to surgical and procedural preference. The third question the anesthesia provider should decide upon is whether needle-based local anesthetic blocks should be instituted or topical airway anesthesia will be the sole technique. Doyle mentions the utility of an antisialogogue as the fourth decision point, and which sedation techniques, if any, as the fifth question. Lastly, the anesthesia provider should decide on the method of tracheal intubation: direct laryngoscopy, flexible bronchoscopy, or video laryngoscopy.

Local anesthetics

The success of ATI is heavily dependent on appropriate airway topicalization. The incidence of epistaxis is reduced with topical vasoconstrictor usage in the nares prior to nasotracheal intubation. A more favorable cardiovascular and systemic toxicity risk profile makes lidocaine a better option as opposed to bupivacaine or ropivacaine. Clinical levels of toxicity have been shown with lidocaine doses greater than 6.0-9.3 mg/kg lean body weight [7]. The total dose of local anesthetics must be considered, including regional anesthesia and surgical infiltration. Although lower concentrations may be just as effective, more rapid onset of anesthesia is associated with higher concentrations.

Practitioners should be wary of benzocaine use, the main component of cetacaine, which can be complicated by methemoglobinemia. Patients with methemoglobinemia exhibit chocolate-colored blood, cyanosis, low pulse oximeter readings, and normal partial pressure of oxygen (PO2) on arterial blood gas. Methylene blue is the first-line treatment. Cocaine, the only local anesthetic with vasoconstrictor properties, is useful for topical anesthesia of the highly vascular nasopharynx. It is available as a solution or a paste, and the maximum recommended dose is 1.5 mg/kg, although caution must be utilized in patients with hypertension, pseudocholinesterase deficiency, and coronary artery disease [6].

There can be variable local anesthetic (LA) absorption based on the delivery device utilized, including but not limited to mucosal atomization, the spray-as-you-go (SAYGO) technique, transtracheal injection, and nebulization [8]. There is insufficient evidence to recommend one technique being superior to the others. There is evidence to suggest that blocks for ATI (glossopharyngeal and superior laryngeal nerve blocks) are associated with higher plasma concentration of LA, increased incidence of local anesthetic systemic toxicity (LAST), and reduced patient comfort [9]. An alternative model for the LA mechanism of action by Scheuer et al. suggests that the prevention of current flow through sodium channels is accomplished via the introduction of a positive charge that electrostatically impedes the flow of sodium ions, rather than directly blocking voltage-gated sodium channels [10].

Although some researchers have suggested dosages as high as 7-9 mg/kg for topically applied lidocaine during ATI without complications; such dosages should likely be avoided in routine clinical practice [11-13]. There continues to be unpredictability in plasma lidocaine levels due to variable pharmacokinetics and various other patient-related factors [14]. Patients who exhibit rapid absorption, impaired liver function, low cardiac output states, or reduced hepatic blood flow are more likely to suffer from LA toxicity with even recommended drug dosages [15]. Martin et al. noted that 92% of subjects that used 2% lidocaine via the SAYGO technique reported central nervous system side effects [11]. The literature also reports death from LA toxicity in a healthy volunteer after fiberoptic bronchoscopy for research purposes [16].

Local anesthetic systemic toxicity

The American Society of Regional Anesthesia and Pain Medicine has published guidelines for the management of LAST [17]. There can be a spectrum of local anesthetic complications ranging from mild symptoms to major central nervous system (CNS) or cardiac toxicity. A number of factors influence the severity and incidence of LAST, including patient characteristics, block technique and location, concomitant medication usage, type and dose of local anesthetic, and timeliness of recognition and provider intervention. Classic symptoms of LAST usually begin with CNS excitement such as circumoral numbness, auditory changes, metallic taste, and agitation. Symptoms can then progress to seizures, CNS depression, coma, and respiratory arrest.

Classically, CNS toxicity precedes cardiac toxicity. When LAST is caused by direct intravascular injection, specifically into the carotid or vertebral arteries, patients can present primarily with seizures, hypertension, tachycardia, and ventricular arrhythmias. In such instances, cardiac depression may quickly follow initial cardiac excitation, resulting in bradycardia, asystole, and hypotension. It is important to note that there is a variability of the LAST presentation, including the onset and duration of symptoms and manifestations. Practitioners should prioritize airway management, cardiopulmonary support, seizure termination, cessation of local anesthetic administration, and lipid rescue protocol. Local anesthetic-induced seizures should be treated with benzodiazepines, as they are associated with a reduced risk of further myocardial depression [17].

If benzodiazepines are not readily available, propofol or thiopental can be utilized to circumvent seizure progression, but care must be taken to avoid cardiac depression. Cessation of severe hypoxia and acidosis can prevent cardiovascular collapse and enable adequate resuscitation.

Cardiac arrest following LAST requires rapid reestablishment of coronary perfusion pressure to improve myocardial contractility and washout of LA. Local anesthetic-induced cardiac arrest is caused by and treated differently than a typical cardiac arrest treated with advanced cardiac life support guidelines. In LA-induced arrest, a standard one-milligram adult dose of epinephrine would be highly arrhythmogenic in a patient already primed for dysrhythmias. For this reason, lower doses of epinephrine are indicated to treat such patients. On the basis of animal studies, vasopressin should be avoided in these patients [17]. Although there is no consensus as to the appropriate dose of epinephrine or vasopressin during resuscitation, a cardiac arrest should prompt the initiation of lipid emulsion therapy, a 1 μg/kg dose of epinephrine, chest compressions, and ventilation with 100% oxygen. In cases of LAST that are refractory, cardiopulmonary bypass should be considered. Patients should be monitored for at least 24 hours following LAST, as depots of local anesthetic can redistribute and lead to delayed recurrence.

Lipid emulsion therapy was first described in rats with bupivacaine toxicity and has been found to be effective in clinical practice [18]. The most popular theory for lipid emulsion therapy is that the emulsion acts as a “lipid sink” that allows lipid-soluble local anesthetics to dissolve into micelles, which are then metabolized by the liver. There is some controversy as to whether to initiate lipid emulsion therapy at the start of mild CNS LAST or wait for cardiovascular LAST.

Sedation

The Standard American Society of Anesthesiology monitors, oxygen via nasal cannula, and capnography should be in place when attempting sedation for ATI, along with an LMA and difficult airway equipment to rescue a lost airway. Yankauer and bronchoscope suction systems should be checked, and adequate intravenous access ensured. Successful ATI requires an adequate level of sedation, in which the patient is able to respond to verbal commands while maintaining spontaneous ventilation and hemodynamic stability. An ideal sedation technique for ATI should include anxiolysis, amnesia, minimal or no recall, appropriate analgesia, and suppression of the cough and gag reflexes. It is imperative to remember that sedation should not be utilized as a substitute for insufficient airway topicalization. In fact, good airway anesthesia may even obviate the need for sedation and enable more successful patient cooperation. Practitioners should be wary of oversedation, whereby respiratory depression, hypoxia, hypercarbia, airway loss, cardiovascular instability, and aspiration can occur. It may be prudent to have an independent anesthesia practitioner monitoring and titrating sedation while another provider attempts ATI. Remifentanil and dexmedetomidine have been associated with a low risk of excessive sedation and high levels of patient satisfaction when utilized for ATI [19]. Propofol, on the other hand, when utilized as a sole sedative agent, is associated with an increased risk of airway obstruction and coughing in comparison to remifentanil [20].

There are a number of classes of medications that can be utilized to enable an adequate plane of anesthesia while maintaining spontaneous ventilation in patients for ATI. Benzodiazepines, opioids, alpha-2 agonists, and intravenous induction agents can be titrated to effect while avoiding respiratory depression and oversedation. There are sparse randomized controlled trials to suggest which sedation technique is best utilized for ATI. 

Airway anatomy

Anesthesia of the upper airway is achieved by blocking sensation to the nasal and/or oral cavities. The trigeminal nerve (cranial nerve V) supplies sensory innervation to the nose while afferent input to the tongue is divided between the lingual nerve (V) supplying the anterior 2/3 of the tongue and the glossopharyngeal nerve (IX) supplying the posterior 1/3. The pharynx receives sensory input from the glossopharyngeal nerve (IX) and the vagus nerve (X). The superior laryngeal nerve (X) innervates the larynx above the vocal cords while the recurrent laryngeal nerve (X) innervates the vocal cords and trachea below. The trachea is supplied by the vagus nerve (X).

Airway blocks

When topical anesthesia has failed or is ineffective for ATI, nerve blocks are indicated, including a glossopharyngeal nerve block, superior laryngeal nerve (SLN) block, and transtracheal block. These blocks are contraindicated in patients with coagulopathies or those who are actively anticoagulated. Aspiration should be instituted prior to LA deposition to avoid intravascular injection, which can lead to nerve injury, seizures, and trauma. Ultrasound can be an excellent tool to increase the success rate of airway blocks by optimizing the accuracy of LA deposition. Ultrasound can be most useful in identifying the greater cornu of the hyoid bone for the SLN block and the cricothyroid membrane for the transtracheal block.

Glossopharyngeal Block

The block anesthetizes the oropharynx by instilling LA along the ninth cranial nerve, which provides sensation to the posterior third of the tongue, the anterior surface of the epiglottis, the vallecula, the tonsils, and the pharyngeal walls. The two techniques described for this block include intraoral and peristyloid [21]. The patient must have adequate mouth opening to utilize the intraoral approach, whereby the tongue is medially retracted with a laryngoscope blade or tongue depressor while LA is injected submucosally at the base of the posterior tonsillar pillars following negative aspiration. Local anesthetic solution is injected at the caudal aspect of the posterior tonsillar pillar, along the crossing of the palatoglossal arch [21].

The peristyloid approach calls for LA infiltration just posterior to the styloid process, at the midpoint of a line from the angle of the jaw to the mastoid process [21]. The needle is inserted perpendicular to the skin, aiming to make contact with the styloid process, after which it is re-angled posteriorly to enable LA deposition. The block can also be performed via direct application of LA-soaked pledgets to the tonsillar pillars or by spraying LA along the nerve’s path. While this method avoids the risk of intravascular injection into the carotid artery, it is typically less successful for a neural blockade. Although this block will abate the gag reflex, it cannot be used as the sole technique for ATI.

Superior Laryngeal Block

**Figure 1:**
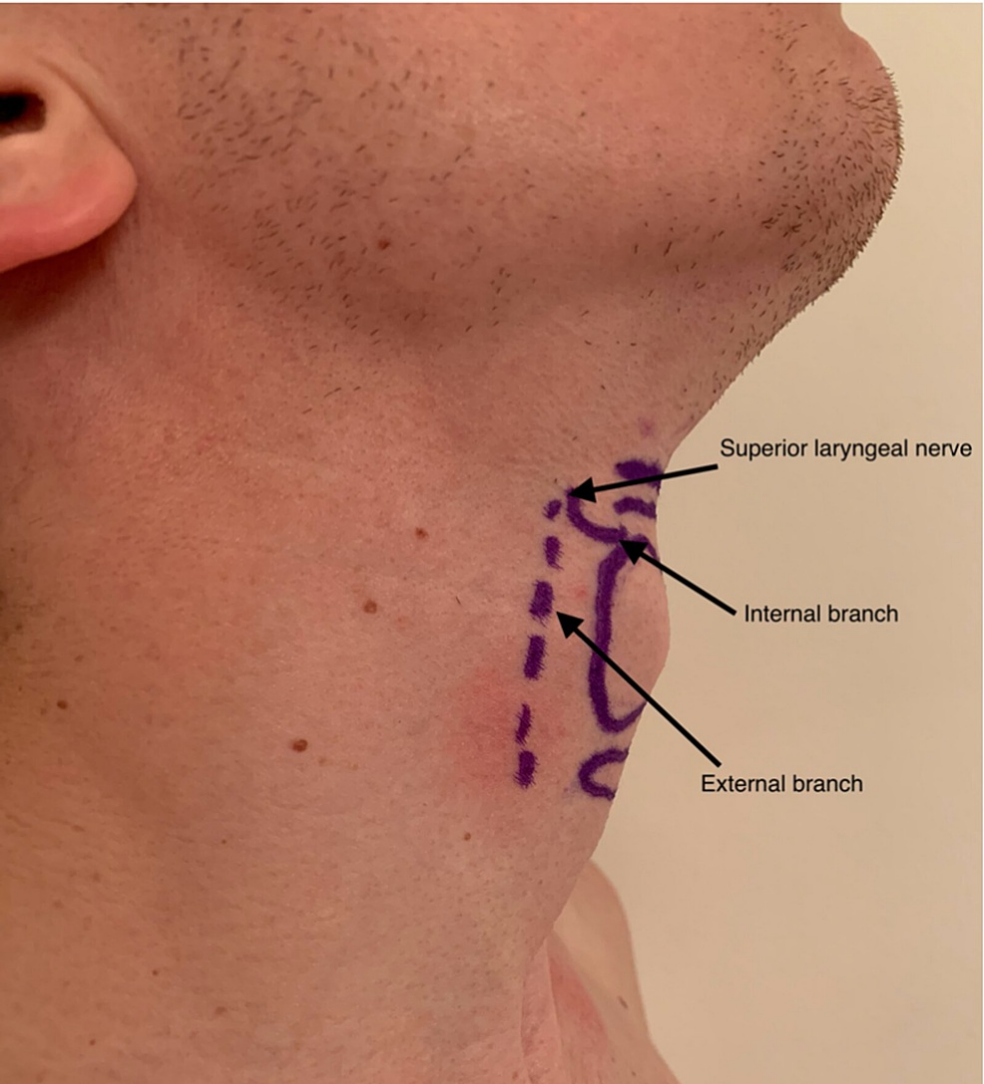
Superior laryngeal nerve anatomy

The internal branch of the SLN provides sensory innervation to the tongue base, the posterior surface of the epiglottis, the arytenoids, and the aryepiglottic folds. This block anesthetizes the larynx above the vocal cords. Anatomically, the internal branch originates from the SLN lateral to the greater cornu of the hyoid bone until it pierces the thyrohyoid membrane. At the greater cornu of the hyoid bone, the SLN splits into the internal and external branches. The internal branch travels submucosally in the piriform recess after it penetrates the thyrohyoid membrane. The external branch travels deep to the sternohyoid muscle (Figure 1).

There are two approaches to blocking the SLN - the internal and external approaches. Utilizing the external approach, the hyoid bone is displaced laterally as a 25-gauge needle is inserted externally, aiming toward the greater cornu. Following contact, the needle is walked off inferiorly and LA is injected to block both the internal and external branches. Careful aspiration is essential, as the path of the carotid artery is nearby [22]. In a patient whose hyoid bone is difficult to isolate, the superior cornu of the thyroid cartilage can be located via identification of the thyroid notch. A needle is inserted at the superior cornu of the thyroid cartilage, then walked cephalad as a local anesthetic is injected. The internal approach for the SLN block utilizes LA-soaked pledgets or gauze placed in the piriform fossae for at least 5-10 minutes. This block also cannot be utilized as the sole technique for ATI.

A newer approach describes the use of a single midline injection for bilateral SLN block [23]. The anterior approach utilizes bilateral injections into the thyrohyoid membrane (THM) 1/3rd of the way from the midline to the lateral edge. This approach avoids neurovascular injection and uncomfortable hyoid bone displacement but requires the injection of 3 ml of LA at two different sites. Fowler et al. describe a novel technique, with a single injection of 6 ml of LA directly into the midline of the THM in order to block bilateral branches of the SLN.

Transtracheal Block

The transtracheal block anesthetizes the recurrent laryngeal nerve (RLN), which provides sensation to the trachea below the level of the vocal cords. The RLN provides motor innervation to all intrinsic muscles of the larynx except the cricothyroid, supplied by the external branch of the SLN. Direct block of the RLN puts the patient at risk for bilateral vocal cord paralysis and airway obstruction, as both sensory and motor fibers travel in tandem. The thyroid cartilage is palpated with the patient’s neck extended while the cricoid cartilage is identified just caudad.

**Figure 2:**
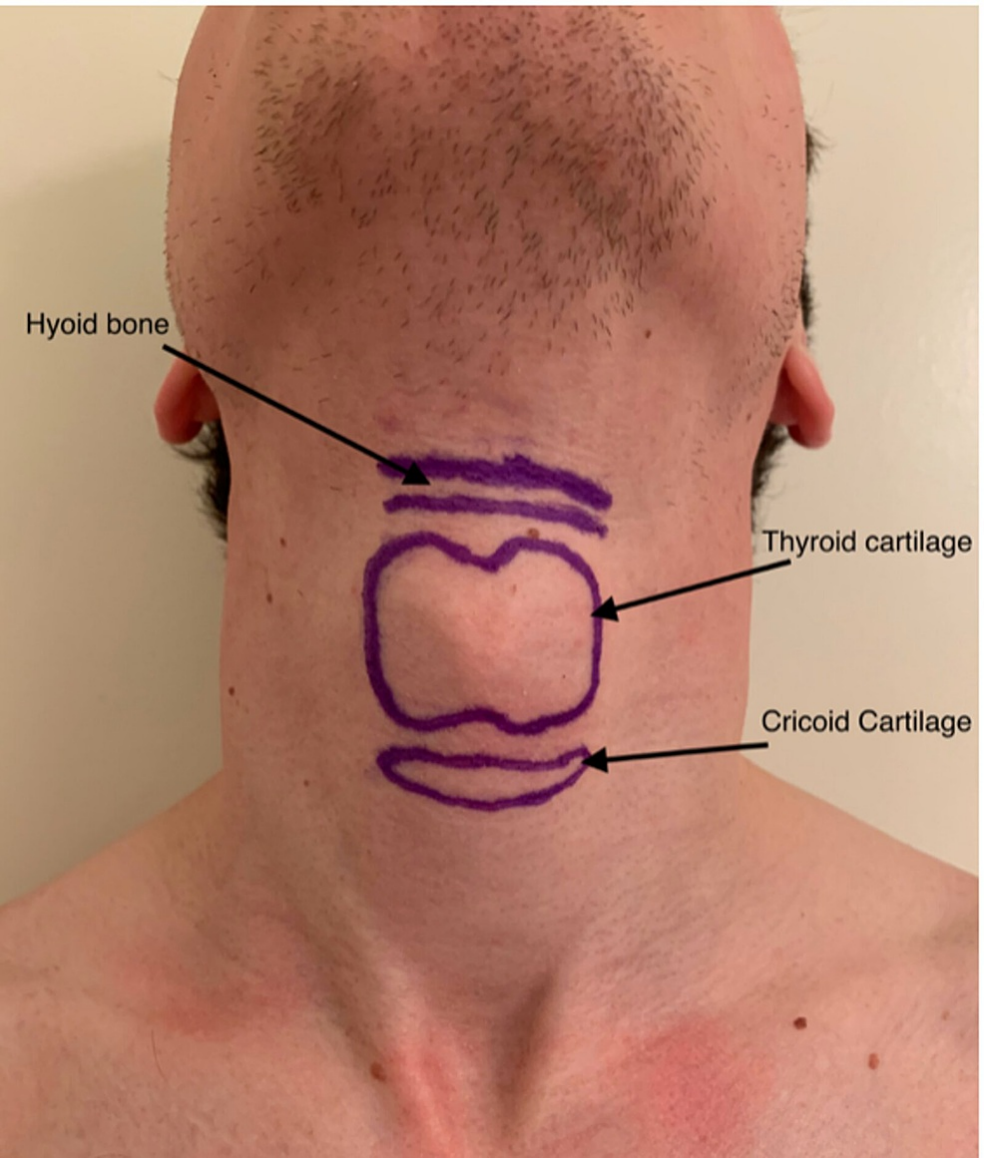
Landmarks for transtracheal block

The cricothyroid membrane lies between these two structures, just superior to the cricoid cartilage (Figure 2). A 22-gauge intravenous catheter is injected through the cricothyroid membrane until air is aspirated while attached to a syringe filled with 4% lidocaine. It is important to stop advancing the needle once tracheal positioning is confirmed, as puncture of the posterior laryngeal wall should be avoided. Continuous aspiration of the syringe is important, as air bubbles offer confirmation of proper tracheal placement. Local anesthetic is then injected as coughing facilitates lidocaine distribution to ensure the blockade of the RLN.

Airway topicalization

**Figure 3:**
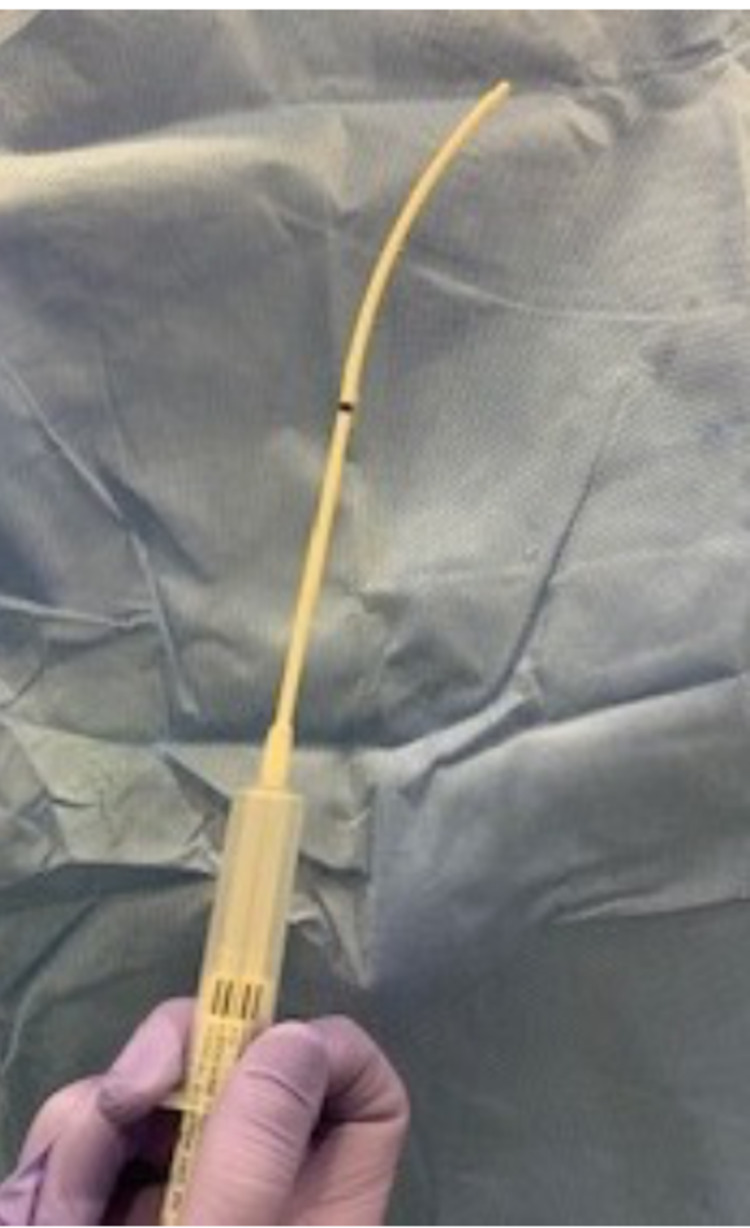
Four percent (4%) lidocaine laryngotracheal anesthesia with a cannula (Abbott Laboratories, Chicago, Illinois)

**Figure 4:**
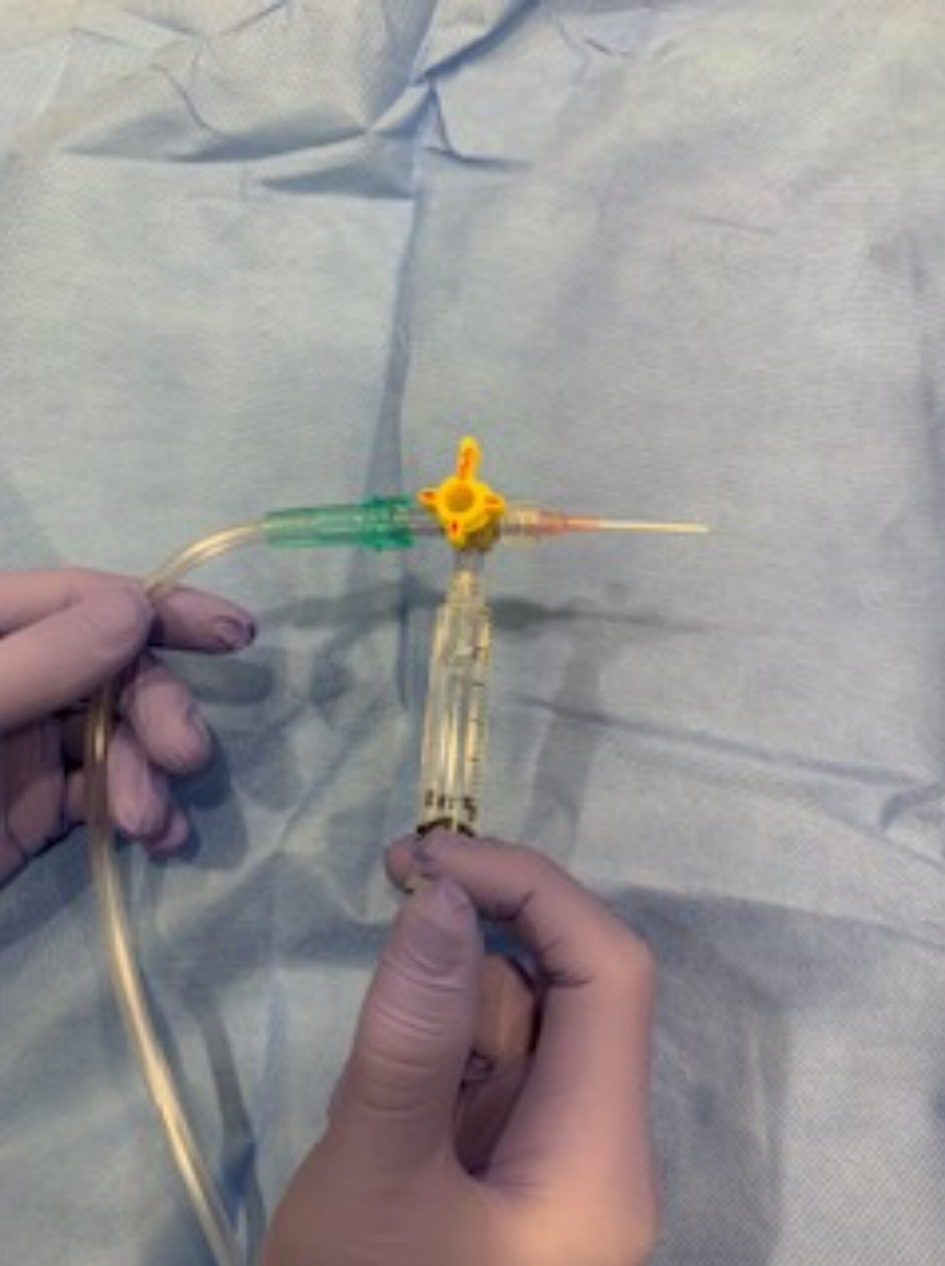
The McKenzie technique for airway topicalization, including a 5 mL syringe containing 4% lidocaine, a 3-way stopcock, 20 G IV angiocath, and oxygen tubing connected to high-flow O2 for the atomizing effect

In addition to airway blocks, there are various techniques to topicalize the upper airway in preparation for ATI. Airway reflexes including coughing, gagging, and laryngospasm, should be suppressed for successful airway management. Atomized lidocaine is frequently utilized to anesthetize the oropharynx. Four percent (4%) lidocaine provides appropriate topical anesthesia at a total dose of 3-4 mg/kg to avoid LAST [18]. Four percent (4%) lidocaine laryngotracheal anesthesia (LTA) with a cannula (Abbott Laboratories, Chicago, Illinois) can be used as an intubation adjunct to probe and topicalize the airway during direct laryngoscopy or video laryngoscopy (Figure 3). Nebulized lidocaine can also be utilized but has a higher risk of pulmonary bronchi absorption, leading to potential toxicity. Topical anesthesia with tetracaine and benzocaine is avoided due to the risk of methemoglobinemia. Local anesthetic can be directly sprayed onto the nasopharynx and oropharynx via the McKenzie technique or via a mucosal atomization device (MAD). The McKenzie technique utilizes a 20-gauge cannula attached to oxygen tubing with a three-way stopcock. The oxygen tubing is attached to an oxygen source, which delivers a flow of 2-4 L/min. As the LA is administered via a syringe, a jetlike spray is created to enable effective topicalization of the nasal and oral mucosa (Figure 4).

**Figure 5:**
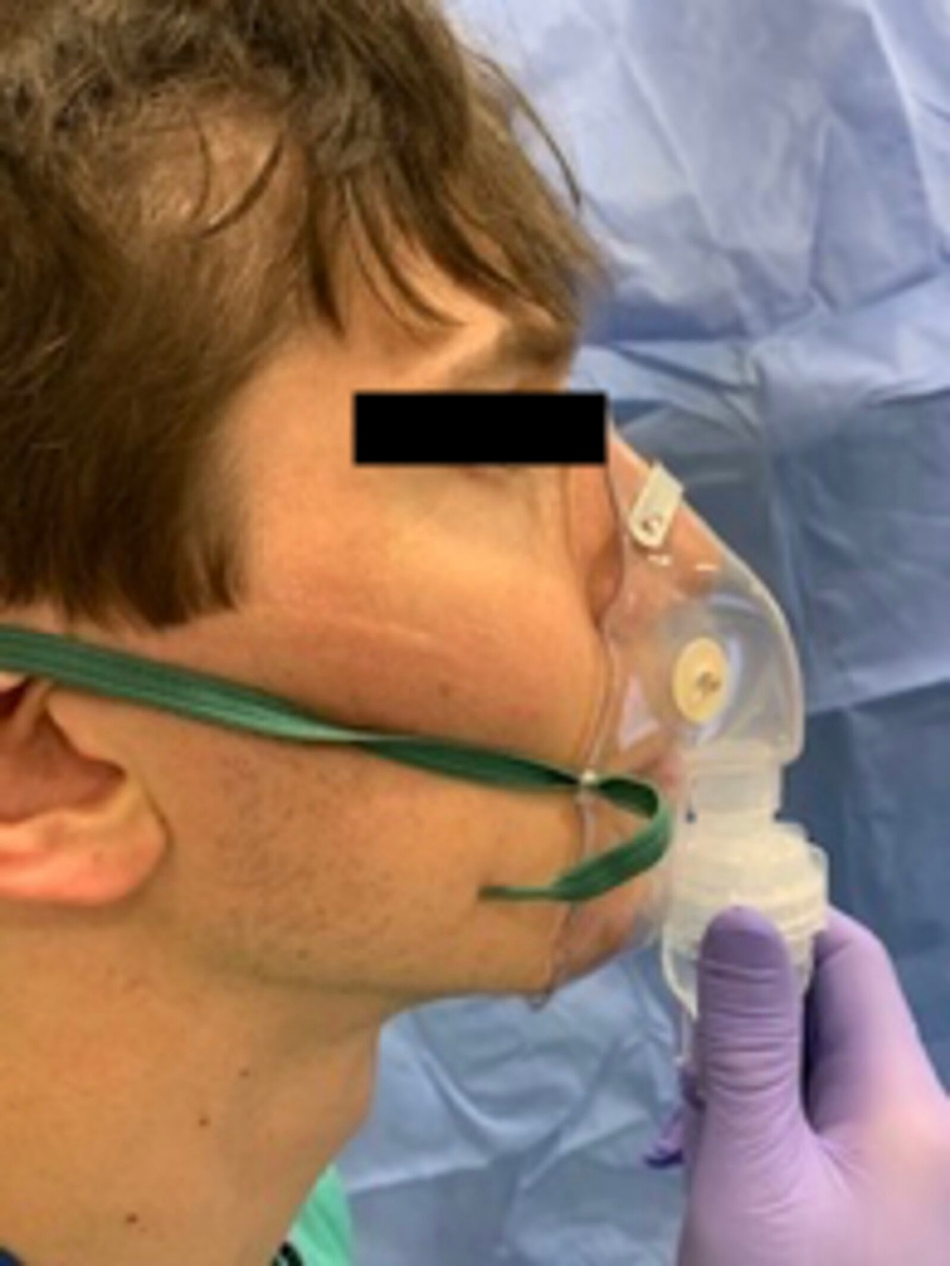
Lidocaine nebulizer including a non-rebreather mask with the bag removed, 10 mL 4% lidocaine in the nebulizer chamber, and a high-flow oxygen tubing connection

Patients can gargle 2% viscous lidocaine, utilize lidocaine paste, or lidocaine-soaked pledgets. In addition, 4% lidocaine can be administered to glottic and oropharyngeal structures using an oxygen-driven power sprayer [23]. The MADgic® laryngotracheal MAD or other commercial devices can also be used for lidocaine administration. The MADett® device can instill LA directly into the lungs via an ETT without interruption to ventilation. As an alternate technique, 4% lidocaine can be added to a nebulizer and delivered with oxygen for 30 minutes as an effective and noninvasive way for airway topicalization down to the trachea in patients with limited mouth opening (Figure 5).

For a nasotracheal intubation approach, local vasoconstriction is essential to reduce the risk of epistaxis, which can further complicate successful airway instrumentation. Although cocaine has been used in the past, its use carries a risk of myocardial ischemia due to coronary artery vasospasm and has largely been replaced by lidocaine and phenylephrine with similar rates of efficacy [24]. Oxymetazoline, a pure α-adrenergic agonist, produces vasoconstriction of the nasal mucosa to prevent epistaxis but has no local anesthetic properties.

**Figure 6:**
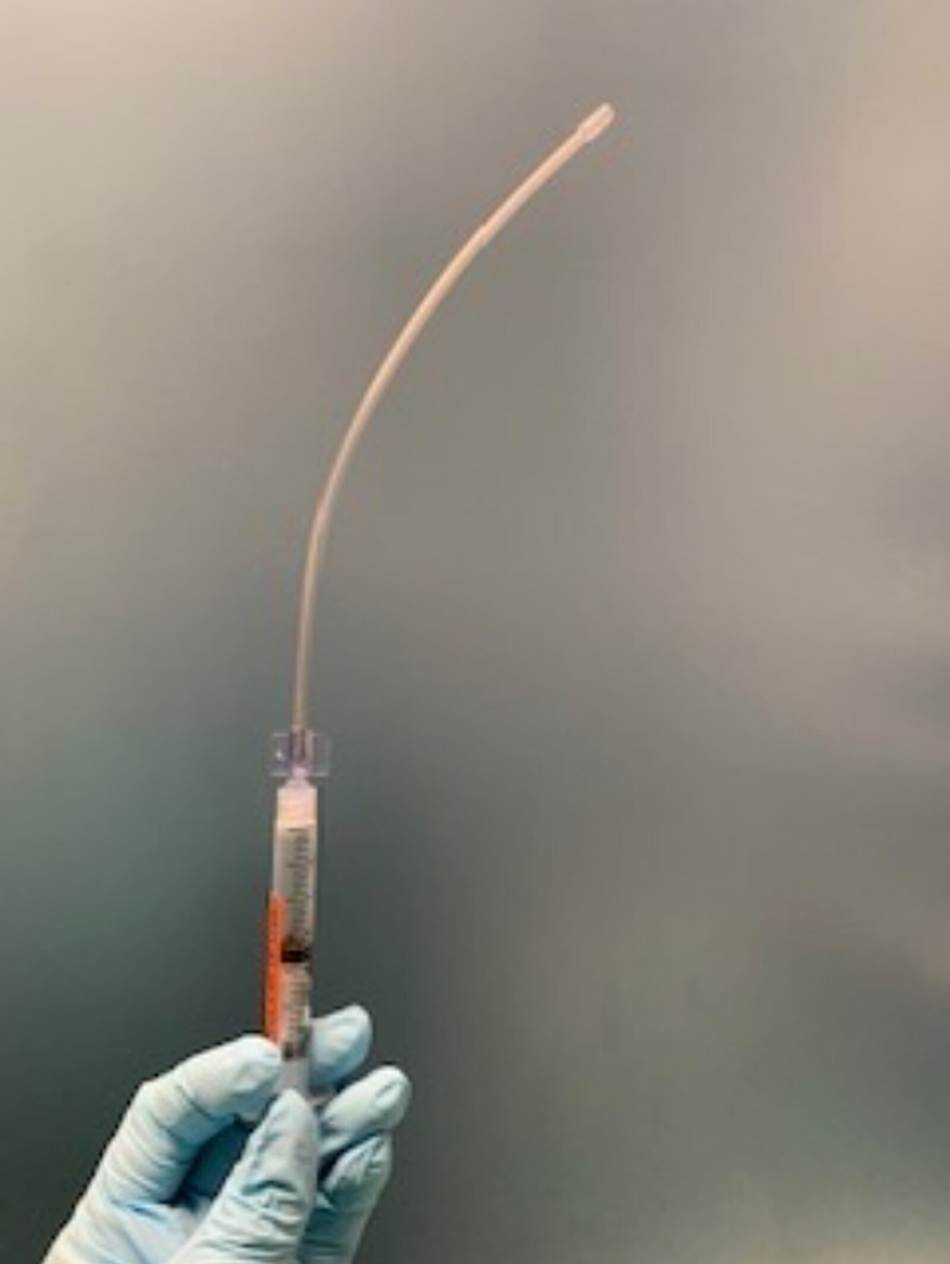
Three (3) mL syringe with 4% lidocaine connected to an atomizer for spray-as-you-go airway topicalization

**Figure 7:**
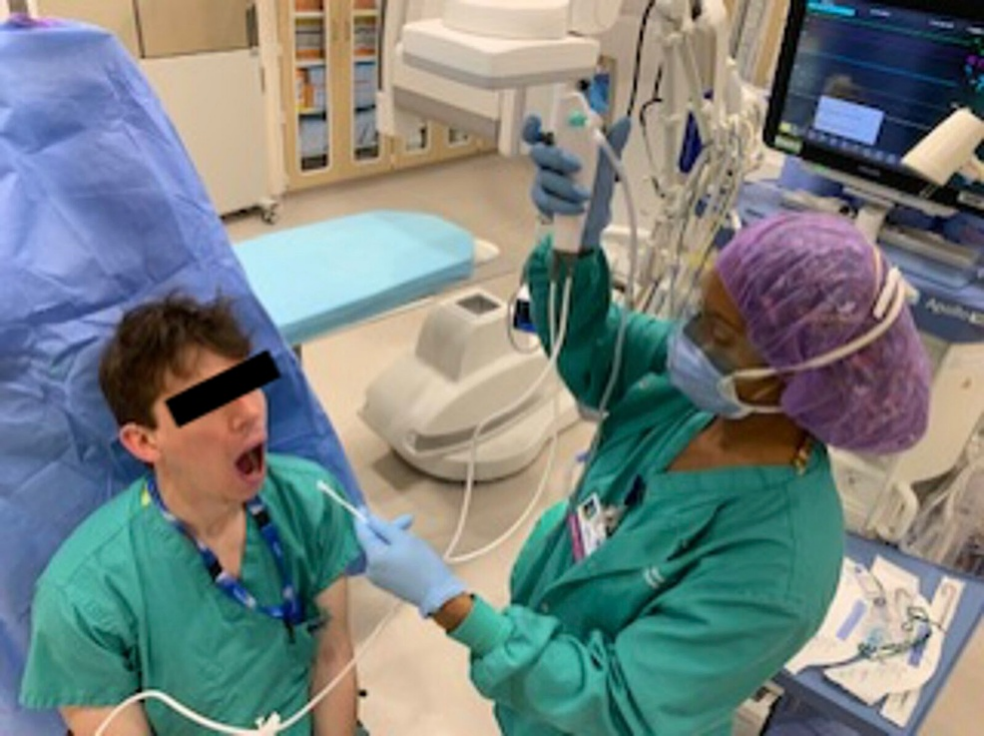
Flexible bronchoscopy for awake intubation with disposable Ambuscope™

The vocal cords can be directly sprayed with LA utilizing the SAYGO technique, as an epidural catheter is inserted through the flexible bronchoscope channel and instilled with LA (Figure 6). With the distal end of the catheter exiting the flexible bronchoscope, LA is placed onto the vocal cords under direct visualization. Utilization of an epidural catheter through the suction channel allows precise application of LA to the desired parts of the airway. Once the vocal cords are sprayed, the solution can also be instilled into the trachea following passage through the rima glottidis. This is especially useful for patients with elevated intracranial pressure, open globe injuries, or unstable cervical spine injuries in whom coughing, bucking, or retching should be avoided. A disposable AmbuScope™ can also be utilized for ATI, enabling flexible bronchoscopy (Figure 7).

A study by Muller et al. compared nebulized lidocaine to topical lidocaine given via a syringe in patients requiring diagnostic bronchoscopy [25]. Administration via a nebulizer was associated with reduced lidocaine dose, improved oxygenation during the procedure, superior cough suppression, and a better safety profile compared to lidocaine instilled via a syringe, although there was no difference in the amount of drug required for sedation for both techniques [25]. In a patient where non-atomized lidocaine is utilized, the LA spread is achieved mainly through the patient coughing immediately following injection. A study by Webb et al. demonstrated more effective airway analgesia with a translaryngeal injection of lidocaine as opposed to the SAYGO technique [26].

A study by Xue et al. compared the safety and efficacy of 2% and 4% lidocaine to provide topical airway anesthesia for patients undergoing fiberoptic bronchoscopy with the SAYGO technique [27]. Following adequate sedation, an oral airway was inserted following jaw lift by an assistant, the flexible bronchoscope was positioned at the epiglottic vallecula near the piriform recess where LA was placed and the scope removed. After five minutes, the procedure was repeated with the drug instilled into the larynx. This procedure was repeated until patients did not exhibit a laryngeal response to LA administration. The flexible bronchoscope was then advanced into the trachea 2 cm below the vocals cords, where more LA was sprayed. Researchers found that both LA concentrations have similar efficacy for topical airway anesthesia utilizing the SAYGO technique, but the total dosage of LA and plasma concentration was lower in the 2% lidocaine group. The authors claim that one of the limitations of using an epidural catheter for LA delivery is the lack of aerosolization, which enables airway mucosa penetration for complete airway topical anesthesia [28].

Airway topicalization with in-circuit nebulized lidocaine has been described. With this technique, the patient underwent an inhalation induction with sevoflurane and then 4% lidocaine was utilized via nebulization with an auxiliary oxygen source. This technique has been successfully described in a pediatric patient with difficult airway and burn injuries by Tsui et al. [29]. This group conducted an in-vitro study to ascertain the rate at which lidocaine is nebulized at different oxygen flow rates. They found that 0.2 mL/min of 4% lidocaine was nebulized with a dispersing oxygen flow rate of 4 L/min from the auxiliary oxygen supply. This 4L/min auxiliary oxygen supply combined with a 4 L/min flow of oxygen and 8% sevoflurane from the anesthesia machine resulted in 8 L/min total gas flow with a 4% concentration of sevoflurane. In the five minutes that this flow was utilized, the patient received 40 mg of lidocaine (1 mL of 4% lidocaine), significantly less than the toxic dose of 4 mg/kg [30].

For the pediatric airway, the most frequently described method for airway topicalization is spraying viscous lidocaine onto laryngeal and tracheal mucosa under direct visualization. The devices utilized either deliver a jet of solution or produce fine, atomized spray [31]. Devices that deliver a local anesthetic jet include Xylocaine 10 mg spray (AstraZeneca UK, Luton, Bedfordshire, UK), the Laryngojet (Amphastar Pharmaceuticals, Rancho Cucamonga, CA), and the Cass needle (Victor-G & Co, Kanpur, India). A metered spray device, the Xylocaine spray delivers a 10 mg dose of viscous lidocaine using a spray nozzle. The Laryngojet and Cass needles enable wider coverage; the former has a prefilled vial of 4% viscous lidocaine attached to a cannula with holes along its entire length to allow circumferential spray. The Cass needle also enables wide coverage due to its four-side openings and distal outlet.

**Figure 8:**
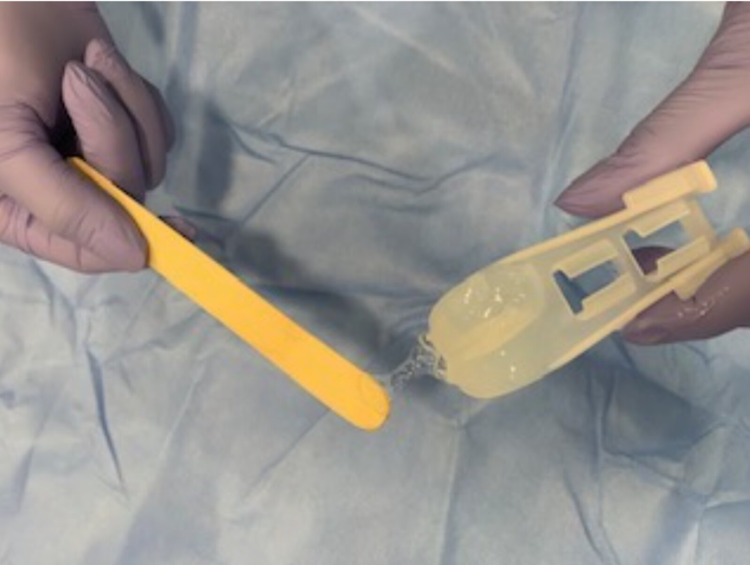
Ovassapian intubating airway coated with 4% lidocaine jelly for topicalization

The MAD® Mucosal Atomization Device (Wolfe Tory Medical Inc., South Salt Lake City, UT) is a flexible stylet attached to a syringe via a fine mist, less likely to irritate the airway than a jet. This technique is useful to anesthetize important laryngeal structures and can only be done under direct laryngoscopy. Although the blind placement of viscous lidocaine into the oropharynx has been shown to anesthetize appropriate laryngeal structures, it is less likely to be effective for the subglottic region [32]. The disadvantages of nebulization are the prolonged time for administration and inaccuracy of the total dose of LA administration due to egress around the mask. Other methods of topicalization include direct administration into the endotracheal tube and application of viscous lidocaine to a laryngeal mask airway or Ovassapian airway (Figure 8).

Table 1 provides a literature review of the techniques available for airway topicalization. 

**Table 1:** Studies reviewed regarding techniques available for airway topicalization

Group	Title	Study Design	Findings
Mostafa et al, 1998 [33]	Nebulized 10% lignocaine for awake fiberoptic intubation	Observational; n=16; pts underwent awake fiberoptic nasal intubation; topical 4% cocaine nasally and 10% lidocaine nebulization	All pts tolerated intubation. No significant change in hemodynamic parameters. Plasma lidocaine remained below the toxic level.
Hai et al, 2011 [34]	A pilot study of the effect of pressure-driven lidocaine spray on airway topical anesthesia for conscious sedation intubation	Observational; n=30; pts were sedated uniformly based on a standardized sedation scale; airway topicalized with 2% lidocaine spray at 10 L/min	All pts intubated on the first attempt; Average intubation condition score 7(+/-1.1) (<10 considered satisfactory); MAP/HR increased significantly following intubation but returned to baseline within 1 minute; No significant changes in SpO2; No adverse reactions/complications
Woodruff et al, 2010 [35]	Atomized lidocaine for airway topical anesthesia in the morbidly obese: 1% compared with 2%	Randomized control trial; n=21; Focus on morbidly obese pts BMI>50; pts received either 40ml atomized lidocaine 1% or 2% with high O2 flow as the carrier	Patients receiving lidocaine 1% had a longer mean time from the start of topicalization to tracheal tube cuff inflation than those receiving lidocaine 2%; 1% cohort demonstrated increased responses to airway manipulation and lower bronchoscopist’s satisfaction scores; No significant difference in hemodynamic responses between groups; Peak plasma conc. lower in 1% group
Xue et al, 2009 [27]	Spray-As-You-Go Airway Topical Anesthesia in Patients with a Difficult Airway: A Randomized, Double-Blind Comparison of 2% and 4% Lidocaine	Randomized control trial; n=52; All patients received 10% lido neb to posterior pharynx/fentanyl and midazolam for sedation; randomly assigned to receive 2% or 4% lidocaine spray as you go; Double-blinded	The total dosage of lidocaine and peak lidocaine concentration were lower in the 2% group; No significant differences in all other variables between groups.
Xue et al, 2007 [36]	Topical anesthesia of the airway using fiberoptic bronchoscope and the MADgic® atomizer in patients with predicted difficult intubation	Observational; n=15; All pts had predicted difficult airway; All received topical anesthesia of oropharynx with “traditional spray techniques”; sedation with fentanyl and midazolam; MADgic atomizer used to spray 3cc 2% lidocaine for topicalization of bilateral piriform recesses and epiglottic vallecula then advanced 2cm past vocal cords with spray of additional 2cc 2% lidocaine	Total time for topicalization ranged from 21-28 min; Median dose of lido was 2.5mg/kg; All pts found airway spray to be acceptable; patient reaction scores were 1.9 ± 0.6 with nasotracheal intubation and 1.6 ± 0.7 with orotracheal intubation
Wang et al, 2019 [37]	1% Tetracaine hydrochloride injection pure solution aerosol inhalation combined with oral administration of dyclonine hydrochloride mucilage as upper airway anesthesia for bronchoscopy: A randomized controlled trial	Randomized control trial; n=523; group A received 1% tetracaine aerosol combined with dyclonine hydrochloride, and group B received diluted 1% tetracaine with dyclonine hydrochloride	Patient’s in Group A tolerated fiberoptic bronchoscope passage better than those in Group B, and their vital signs were more stable throughout; 3 pts in Group A reported numbness in hands/feet after atomization of 1% tetracaine
Khandelwal et al, 2018 [38]	Role of lignocaine nebulization as an adjunct to airway blocks for awake fiber-optic intubation: A comparative study	Randomized control trial; n=60; One group received 2% lidocaine neb and the other group got a placebo with NS neb. Both groups of patients proceeded to receive bilateral superior laryngeal and transtracheal blocks	Statistically, no significant differences were found in hemodynamic parameters, demographics, intubation time, and intubation grading scale in both groups; However, overall patient comfort and satisfaction score was better in the group that received a combination of nebulized lidocaine and blocks
Vasu et al, 2017 [39]	Efficacy of atomized local anesthetic versus transtracheal topical anesthesia for awake fiberoptic intubation	Randomized control trial; n=33; Patients either received transtracheal injection of 4% lidocaine or 4% atomized lidocaine DeVilbiss atomizer prior to awake fiberoptic; All patients received 10% lidocaine spray and 2% lidocaine jelly to each nostril prior	Ease of intubation, patient comfort, and the time taken to intubate were significantly better in the group receiving transtracheal lidocaine; No significant changes in hemodynamic parameters
Webb et al, 1990 [26]	Local anesthesia for fiberoptic bronchoscopy: Transcricoid injection or the “spray as you go” technique?	Randomized control trial; n=62; Patients were adults presenting for routine diagnostic fiberoptic bronchoscopy; All pts received 5cc 2% lidocaine gel to each nostril; 32 pts were assigned to 4% lidocaine spray group and 30 pts assigned to transcricoid puncture with 5cc 2% lidocaine	Pts receiving transcricoid puncture coughed less and required less total lidocaine; No significant difference in pt perception of either approach
Venkatnarayan et al, 2020 [40]	Comparison of spray catheter with the 'spray-as-you-go' technique for airway anesthesia during flexible bronchoscopy - A randomized trial.	Randomized control trial; n=130; Patients undergoing bronchoscopy were randomized to receive airway anesthesia with 2% lidocaine through a spray catheter or the 'spray-as-you-go' technique through the working bronchoscope channel	Requirement for sedation was lower in the spray catheter group; less coughing and increased operator satisfaction in spray catheter group; patient satisfaction not statistically different between the two groups
Müller et al, 2018 [25]	Nebulization versus standard application for topical anesthesia during flexible bronchoscopy under moderate sedation – a randomized controlled trial	Randomized control trial; n=60; Patients were randomized to lidocaine application via syringe or nebulizer; all pts received a midazolam bolus, and further administration of propofol and fentanyl was at provider’s discretion to help with pt tolerance of bronchoscope	Pts required lower doses of end-bronchial lidocaine in neb group; no differences in the dosage of sedative drugs were observed between the two groups; pts in neb group had higher mean O2 sat on ABG and lower complication rate
Hamad et al, 2014 [41]	Evaluation of the efficacy of transcricoid lignocaine as adjunctive local anesthesia for fiberoptic bronchoscopy	Randomized control trial; n=50; consisted of inpatients and outpatients for bronchoscopy; All pts received topical 2% lidocaine to airway; pts were randomized to receive 2% lidocaine transcricoid; All pts received midazolam and fentanyl for sedation; operating bronchoscopes carried out the bronchoscopy procedure, unaware of which type of local anesthesia was used on the vocal cords	Significant improvement in the perceived ease of procedure and frequency of coughing during the procedure
Dreher et al, 2016 [42]	Nebulized versus standard local application of lidocaine during flexible bronchoscopy: A randomized controlled trial	Randomized control trial; n=30; Pts requiring bronchoscopy with end-bronchial or transbronchial biopsy were randomized to either receive topical lidocaine via syringe or nebulizer; all pts received midazolam and propofol bolus; propofol infusion rate and fentanyl bolus titrated to pt comfort	Patients in the nebulizer group required lower doses of endobronchial lidocaine and intravenous fentanyl than those in the syringe group; no differences in propofol dosages/pt tolerance/pt safety
Stolz et al, 2005 [43]	Nebulized lidocaine for flexible bronchoscopy: A randomized, double-blind, placebo-controlled trial	Randomized control trial; n=150; pts undergoing diagnostic flexible bronchoscopy were randomized to receive either 4 mL of 4% lidocaine or 4 mL of saline solution as placebo via nebulization; pts sedated with 5 mg of IV hydrocodone and midazolam boluses	Hemodynamic findings, duration of the procedure, cough scores for physicians and patients, discomfort score for patients, midazolam doses, and supplemental lidocaine doses, were similar in both groups; the mean total lidocaine dose in the lidocaine neb group was higher than in the placebo group
Dhasmana et al, 2014 [44]	Nebulization versus spray-as-you-go airway topical anesthesia in patients with temporomandibular joint ankylosis using 2% lidocaine	Randomized control trial; n=60; adult pts with TMJ ankylosis; assigned to either receive 2% lidocaine neb or spray as you go; all pts received midazolam 0.05 mg/kg and fentanyl 2 ug/kg; spray as you go group received 10% lidocaine spray to nasal cavity/nasopharynx	No statistical differences in observed variables
Ho et al, 2020 [45]	Is additional nebulized lidocaine helpful in flexible bronchoscopy?	Meta-analysis; 7 RCTs with 1366 pts included	Between nebulized lidocaine group and no additional nebulizer lidocaine group, measures of cough were no different, as were operator satisfaction score, ease of procedure, and pt discomfort; nebulized lidocaine group required a higher total lidocaine dose
Tsui et al, 2004 [29]	Fiberoptic endotracheal intubation after topicalization with in-circuit nebulized lidocaine in a child with a difficult airway	Case report; 8yM with developmental delay and 65-70% TBSA burn for scar release and skin grafting nasal/facial/neck region. Inhalational induction with 8% sevoflurane; IV glycopyrrolate was given; 4% lido nebulized in-circuit at 4L/min followed by successful fiberoptic intubation	Upper airway manipulations, such as awake fiberoptic intubation, are poorly tolerated in pediatric patients; small-volume nebulizers used in adults would be poorly tolerated in an uncooperative pediatric pt; simultaneous administration of volatile anesthetic with in-circuit nebulized lidocaine provided good conditions for fiberoptic intubation while maintaining spontaneous ventilation

Utility of supraglottic airways and surgical airway management

If initial tracheal intubation fails, the insertion of a laryngeal mask airway (LMA) or intubation LMA (ILMA) is useful to facilitate appropriate ventilation and oxygenation while also providing a pathway for fiberoptic intubation. Fiberscope-assisted intubation through an ILMA has been shown to have a higher success rate than blind intubation [46-48].

Surgical cricothyroidotomy is reserved for emergency airway situations in which patients cannot be adequately intubated or ventilated. In instances where a supraglottic airway cannot be effectively placed due to anatomical airway abnormalities, secretions, or blood, a surgical airway is indicated. The National Emergency Airway Registry found that out of 17,500 adult intubations performed in emergency departments over 10 years, 0.14% underwent a primary surgical airway and 0.31% received a rescue surgical airway, most commonly performed in trauma victims [49]. Relative contraindications to cricothyroidotomy include laryngeal fracture, laryngotracheal disruption, and tracheal transection.

Four commonly used techniques include the bougie-assisted cricothyroidotomy, the standard technique, the rapid four-step, and the Seldinger technique. Ultrasound identification of the cricothyroid membrane is useful in patients for cricothyroidotomy. Complications associated with the procedure are largely dependent on patient co-morbidities, the clinician’s level of training, procedural setting (emergency department, out of the hospital, operating room), and the clinical scenario. Major complications include injury to the thyroid or cricoid cartilage, tracheal rings, tracheal or esophageal perforation, unintentional tracheostomy, subcutaneous emphysema, bleeding, and infection. Long-term complications include dysphagia, subglottic stenosis, and permanent voice changes.

## Conclusions

The safety and efficacy of airway topicalization for ATI is based upon a number of factors, including appropriate sedation, adequate local anesthetic application, utilization of difficult airway equipment, and practitioner skill level and expertise. There remains a lack of high-quality evidence for ideal topicalization techniques, sedation strategies, local anesthetic administration methods, and their subsequent outcomes during ATI. Further investigation is required in order to improve clinical and patient-centered outcomes in patients with a difficult airway. Future steps to optimize ATI include continued training with alternative airway devices, improved capnography and patient monitoring, and improved local anesthetic delivery devices.
